# Analysis of ultrastructural defects in sperm by transmission electron microscopy in asthenozoospermia patients: a study from multiple centers across China

**DOI:** 10.1186/s40364-025-00841-8

**Published:** 2025-10-08

**Authors:** Mo-qi Lv, Hai-xu Wang, Hou-yang Chen, Jun-cheng Gao, Hai-feng Song, Yu-dong Zhang, Tao Li, Xiao-hui Ling, Lin-jie Zhu, Jun-ren Nong, Yu-jiao Tong, Liang-cai Lu, Yun-jie Wen, Yue-xiao Wang, Guang-yu Qin, Jing Li, Guan-chen Liu, Yan-qi Yang, Pan Ge, Nan Zhang, Rui-fang Sun, Ying Sun, Jian Zhang, He-cheng Li, Yi-na Jiang, Ming Liu, Dang-xia Zhou

**Affiliations:** 1 Department of Pathology, School of Basic Medical Sciences, Medical School, Xi’an Jiaotong University, Xi’an, 710061 China; 2 Key Laboratory of Environment and Genes Related to Diseases, Ministry of Education, Xi’an, 710061 China; 3 Assisted Reproduction Center, Xijing Hospital of Air Force Medical University (the former the Fourth Military Medical University), Xi’an, 710032 China; 4 Jiangxi Maternal and Child Health Hospital, Nanchang, 330000 China; 5 Northwest A&F University, Xianyang, 712100 China; 6 Xianyang Central Hospital, Xianyang, 712000 China; 7 Affiliated Hospital of Shaanxi University of Chinese Medicine, Xianyang, 712000 China; 8 Reproductive Center, Baoji Maternal and Child Health Hospital, Baoji, 721000 China; 9 Reproductive Medicine Centre, Huizhou Municipal Central Hospital, Huizhou, 516001 China; 10 First Hospital of Handan City, Handan, 056000 China; 11 Guangxi Zhuang Autonomous Region Ethnic Hospital, Nanning, 530000 China; 12 Health Science Center, Xi’an Jiaotong University, Xi’an, 710061 China; 13 Guangzhou Huayin Medical Laboratory Center Ltd, Guangzhou, 510000 China; 14 School of Life Science and Technology, Xi’an Jiaotong University, Xi’an, 710049 China; 15 Department of Pathology, Hong Hui Hospital, Xi’an Jiaotong University, Xi’an, 710054 China; 16 Department of Urology, Second Affiliated Hospital of Xi’an Jiaotong University, Xi’an, 710001 China; 17 Department of Pathology, First Affiliated Hospital of Xi’an Jiaotong University, Xi’an, 710061 China; 18 Department of Gynecology and Obstetrics, Second Affiliated Hospital of Xi’an Jiaotong University, Xi’an, 710001 China

**Keywords:** Transmission electron microscopy, Sperm ultrastructure, Asthenozoospermia patient

## Abstract

This study aimed to identify the ultrastructural features of sperm in Chinese asthenozoospermia patients and to evaluate their clinical implications. A total of 139 individuals, including 106 asthenozoospermia patients and 33 fertile controls, were recruited from multiple centers across China. Sperm ultrastructural defects were examined using transmission electron microscopy (TEM), while conventional sperm quality was assessed by light microscopy. Compared with the control group, the asthenozoospermia group showed significant ultrastructural defects, particularly in the sperm tail, including mitochondrial and axonemal defects. Based on tail ultrastructure, participants were further categorized into four groups (normal ultrastructure, simple abnormal axonemes, simple abnormal mitochondria, and both abnormality in axonemes and mitochondria). All three abnormal groups indicated significantly lower value in sperm (motile/kinematic) quality compared to the normal group. However, no statistically significant differences in sperm quality were observed among the three abnormal groups. These nationwide findings indicated that TEM could remedy the limitations of conventional light microscopy, which is difficult to localize organelle-specific defects. This capability may ultimately inform individualized diagnostic and therapeutic strategies.

**Supplementary Information:**

The online version contains supplementary material available at 10.1186/s40364-025-00841-8.

To the editor

Asthenozoospermia, a common cause of male infertility, is characterized by reduced or absent sperm motility in fresh ejaculate [1]. Severely impaired motility is often associated with abnormalities in the sperm tails [2], primarily resulting from dysfunction of the flagellar and/or mitochondria [3, 4]. A clear understanding of the specific underlying mechanisms is crucial for developing targeted diagnostic approaches and therapeutic strategies, enabling individualized treatment plans. However, the limited resolution of conventional light microscopy makes it unsuitable for assessing these subcellular organelles. In contrast, transmission electron microscopy (TEM) allows for high-resolution visualization of sperm ultrastructure and the identification of specific organelle defects [5]. Despite this capability, few studies have utilized TEM to investigated the ultrastructural defects in sperm from men with asthenozoospermia. Given this background, our study aimed to characterize sperm ultrastructure in asthenozoospermia patients using TEM.

In this study, we assessed ultrastructural defects of sperm using TEM and analyzed sperm quality using light microscopy or computer automated semen analysis system (CASA) in 139 participants. The detailed study population, questionnaire and physical examination, sperm assay, transmission electron microscopy, and statistical analysis are provided in Supplementary Materials: Methods.

A total of 106 asthenozoospermia patients and 33 controls were included in this study. Clinical characteristics of the patients were summarized in Fig. S1 and Table S1. Ultrastructural performance of sperm was shown in Fig. 1. Compared with control group, asthenozoospermia group showed fewer normal axonemes (46.2%vs72.7%, *P* = 0.008), dense fibers (43.4%vs72.7%, *P* = 0.003), and mitochondria (20.8%vs57.6%, *P* < 0.001) in midpiece of the tail, and showed fewer normal axonemes in both principal piece (25.5%vs60.6%, *P* < 0.001) and endpiece (9.4%vs36.4%, *P* < 0.001) of the tail (Table S2). In neck of tail, asthenozoospermia group had more cytoplasmic residues (87.7%vs72.7%, *P* = 0.039) and fewer centriole (30.2%vs57.6%, *P* = 0.004) when compared with control group (Table S2). In head of sperm, asthenozoospermia group had fewer normal chromatin (34.9%vs63.6%, *P* = 0.003), however, head shape (*P* = 0.789) and acrosomes (*P* = 0.767) exhibited little differences in frequency between asthenozoospermia group and control group (Table S2).

**Fig. 1:**
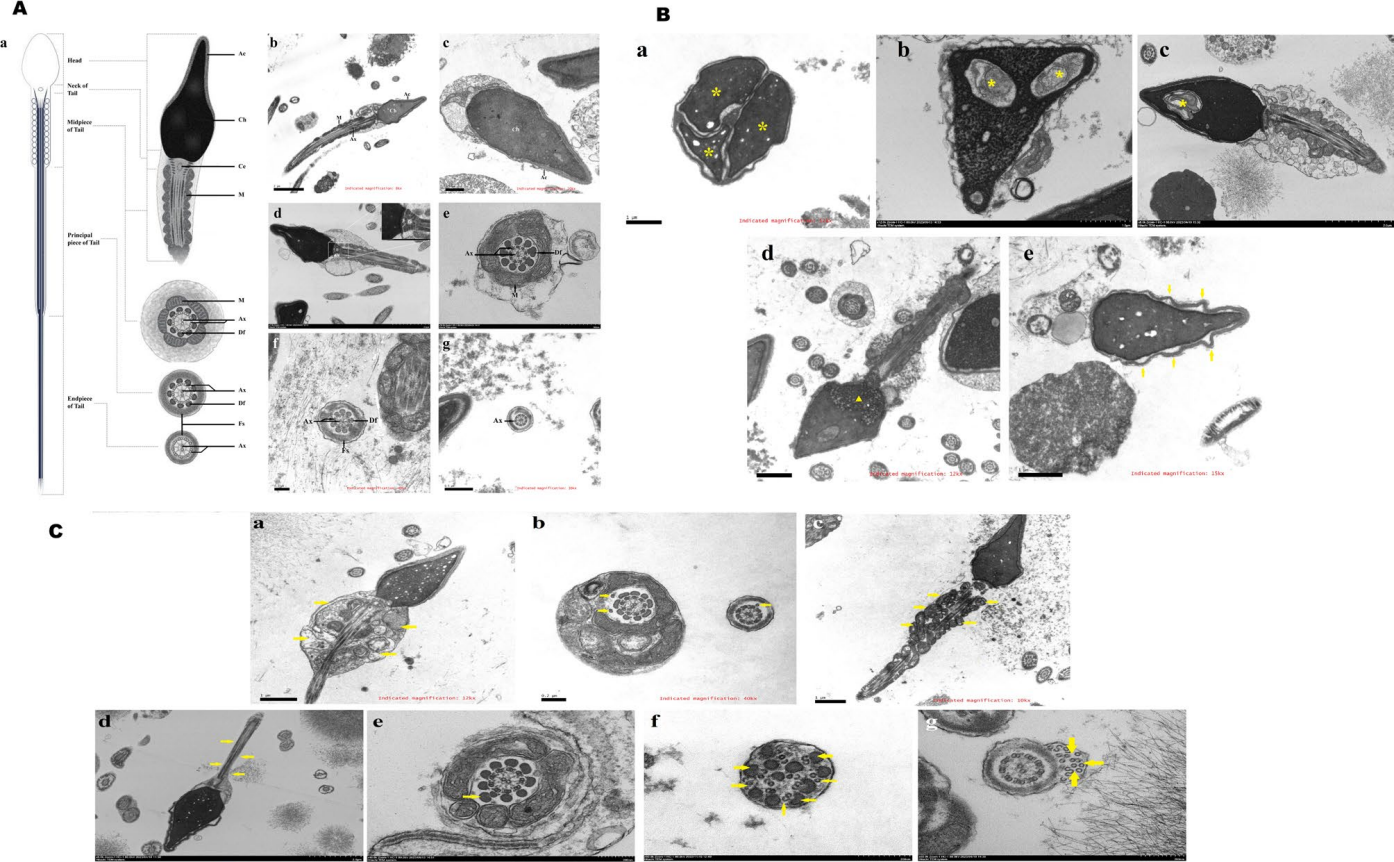
Electron micrographs of typical morphological features of human sperm. (**A**) Electron micrographs of normal morphological features in human sperm: (a) mode diagram of the typical morphological features of human sperm; (b) longitudinal section of human sperm: the normal sperm had a symmetrical mid-piece with smooth axoneme surrounded by regularly arranged mitochondria; (c) sperm head (longitudinal section): normal chromatin condensation (Ch), no vacuoles (simple membrane vacuoles or vacuoles with membrane whorls) or small membrane vacuoles occupying a little part of nucleus, the acrosome covers approximately two-thirds of the head without swelling, and the acrosome membrane is intact (Ac); (d) neck of sperm tail (longitudinal section): the neck region appears conserved, with the well-defined centriole (Ce); (e) midpiece of sperm tail (cross-section), the typical axoneme and peri-axoneme structure: the axoneme mainly comprises a “9 + 2” structure, including nine pairs of peripheral doublet microtubules (AX, upper arrow) and a central pair of microtubules (AX, lower arrow). The peri-axoneme structure includes the nine dense fibers (Df), and helical mitochondria (M); (f) principal piece of sperm tail (cross-section), the typical axoneme and peri-axoneme structure: the axoneme mainly comprises a “9 + 2” structure, including nine pairs of peripheral doublet microtubules (AX, upper arrow) and a central pair of microtubules (AX, lower arrow). The peri-axoneme structure includes the nine dense fibers (Df), and the fiber sheath (Fs); (g) endpiece of sperm tail (cross-section), the typical axoneme structure: the axoneme mainly comprises a “9 + 2” structure, including nine pairs of peripheral doublet microtubules and a central pair of microtubules (AX, lower arrow). (**B**) Electron micrographs of sperm head defects: (a) longitudinal section: trinucleated head (*); (b) longitudinal section: altered shape of the head, severe vacuolar defect of the chromatin (*), granular chromatin; (c) longitudinal section: severe vacuolar defect of the chromatin (*); (d) longitudinal section: partly granular chromatin (triangle); (e) longitudinal section: a displaced acrosomal vesicle (arrow). (**C**) Electron micrographs of sperm tail defects: (a) longitudinal section: cytoplasmic residues (arrow), the midpiece is enlarged with aggregates of misaligned pale mitochondria; (b) cross-section: supernumerary axonemes (arrow) in midpiece; (c) longitudinal section: number of mitochondria is increasing, swollen, irregular in shape (arrow) in midpiece; (d) longitudinal section: mitochondria are absent or mitochondria are scarce (arrow) in midpiece; (e) cross-section: a supernumerary dense fiber (arrow) in midpiece; (f) cross-section: translocation of axonemes and dense fibers, absence of fibrous sheath in principal piece; (g) cross-section: misassembled axonemal structures (arrow) in endpiece. Ac: Acrosomes, Ax: Axonemes (typical structure of “9 + 2” core axoneme), Ce: Centriole, Ch: Chromatin, Df: Dense fibers, Fs: Fibrous sheath, M: Mitochondria

Additionally, ultrastructural performances also showed significant correlations with baseline characteristics and sperm parameter (Fig. S2).

Moreover, considering that recent researches mainly attribute the mechanism of asthenozoospermia to the dysfunction of sperm flagellar and sperm mitochondria [3, 4]. We further divided participants into four groups: normal ultrastructure (NU), simple abnormal axonemes (SAA), simple abnormal mitochondria (SAM), and both abnormality in axonemes and mitochondria (BAAM) (Table S3). Compared with NU, the three abnormal groups demonstrated significant lower value in sperm parameters (Fig. 2). Specifically, compared with NU, BAAM showed significant lower value in total sperm motility (TSM: 32.541 ± 22.129 vs. 52.965 ± 22.711, *P* = 0.003), progressive sperm motility (PSM: 21.422 ± 18.727 vs. 41.620 ± 23.130, *P* = 0.001), velocity average path (VAP: 25.844 ± 15.354 vs. 37.326 ± 15.189, *P* = 0.026), and wobble (WOB: 54.787 ± 21.020 vs. 68.046 ± 9.387, *P* = 0.027) (Fig. 2). Similarly, SAA exhibited significant lower value in TSM (24.700 ± 12.688 vs. 52.965 ± 22.711, *P* = 0.030), PSM (20.270 ± 10.541 vs. 41.620 ± 23.130, *P* = 0.039), and amplitude of lateral head displacement (ALH: 1.367 ± 1.504 vs. 3.926 ± 1.724, *P* = 0.025) (Fig. 2). Meanwhile, SAM showed significant lower value in TSM (33.552 ± 20.090 vs. 52.965 ± 22.711, *P* = 0.005), PSM (23.612 ± 19.038 vs. 41.620 ± 23.130, *P* = 0.004), velocity straight line (VSL: 23.290 ± 12.241 vs. 37.355 ± 27.170, *P* = 0.032), VAP (27.197 ± 11.010 vs. 37.326 ± 15.189, *P* = 0.017), and ALH (2.425 ± 1.484 vs. 3.926 ± 1.724, *P* = 0.004) (Fig. 2). Remarkably, however, no statistic differences of sperm parameters were seen among the three abnormal groups (Fig. 2).

**Fig. 2:**
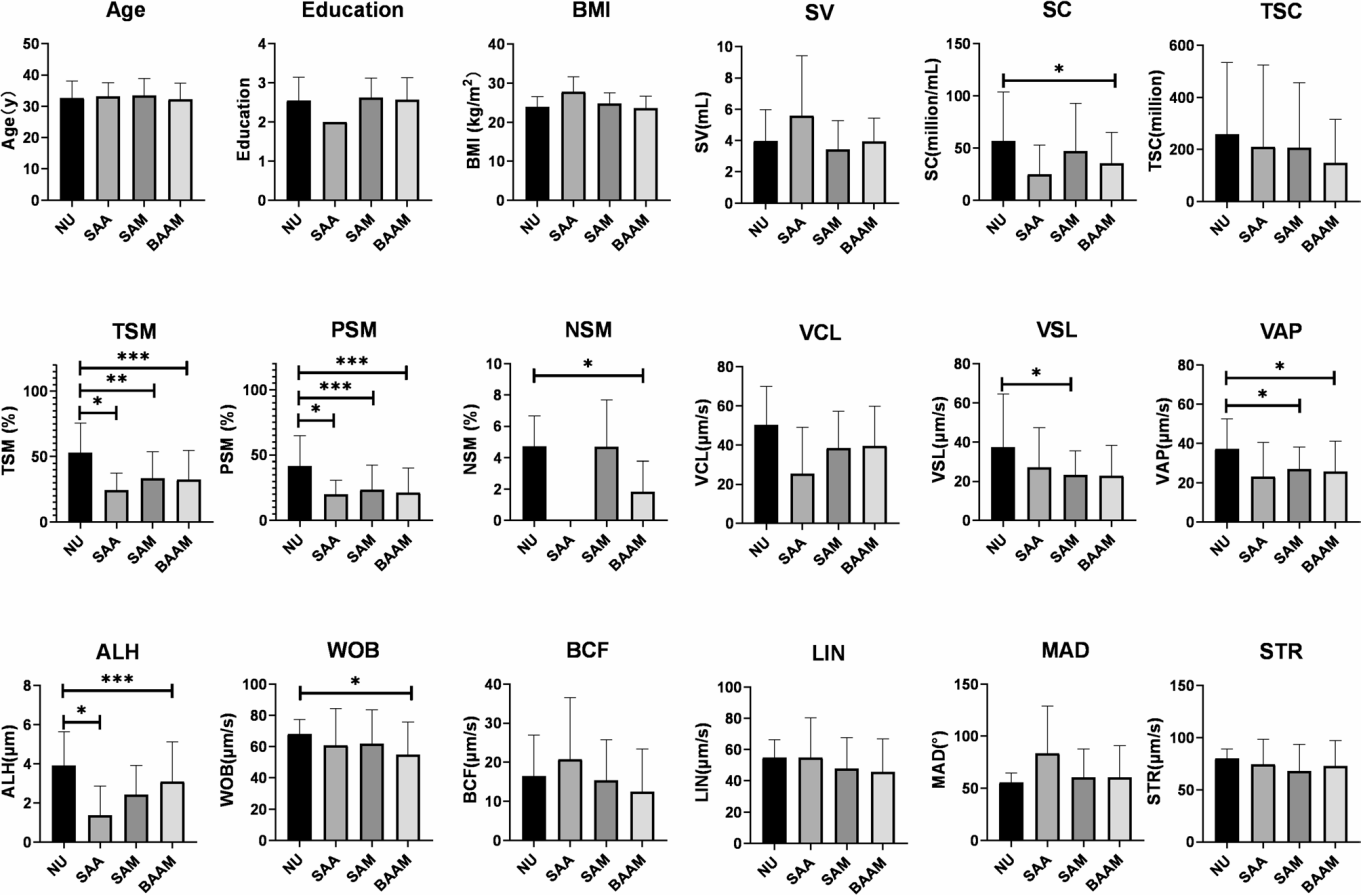
Differences of baseline characteristics and sperm parameters among normal ultrastructure group, simple abnormal axonemes group, simple abnormal mitochondria group, and both abnormality group. ALH, amplitude of lateral head displacement; BAAM, both abnormity in axonemes and mitochondria; BCF, beat cross frequency; BMI, body mass index; LIN, linearity; MAD, mean angular displacement; NSM, normal sperm morphology; NU, normal ultrastructure; PSM, progressive sperm motility; SAA, simple abnormal axonemes; SAM, simple abnormal mitochondria; SC, sperm concentration; STR, straightness; SV, semen volume; TSC, total sperm count; TSM, total sperm motility; VAP, velocity average path; VCL, velocity curvilinear; VSL, velocity straight line; WOB, wobble. *: P < 0.05, **: P < 0.01, ***: P < 0.005.

To the best of our knowledge, this is the first nationwide study in China to report severe sperm ultrastructural defects in asthenozoospermia patients, consistent with studies in other populations [6, 7]. Further analysis demonstrated that, compared with the normal ultrastructure group, the three abnormal groups (SAA, SAM, BAAM) exhibited significantly lower TSM, PSM, and reduced kinematic parameters. However, no significant statistical differences in sperm parameters were observed among the three abnormal groups. This suggested that light microscopy and/or CASA alone cannot distinguish whether impaired motility arises from axonemal or mitochondrial defects.

Assisted reproductive technique (ART), including in vitro fertilisation (IVF) and intracytoplasmic sperm injection (ICSI), are standard treatments for most infertility cases [8]. ARTs are scientifically demanding and personnel-intensive, making them costly [9], yet their success rates remain suboptimal [10]. Consequently, accurately predicting ART outcomes before treatment is critical. A previous Chinese study indicated that mitochondrial, but not flagellar, defects were associated with clinical pregnancy rates [11]. Similarly, two contemporaneous French studies confirmed that flagellar ultrastructural defects do not predict ICSI outcomes [6, 12]. Together, these studies across multiple populations enhance the generalizability of the findings. Other relevant researches were summarized in Table S4. Our findings emphasize the superior diagnostic value of TEM over light microscopy in determining whether impaired motility originates from mitochondrial or axonemal defects. As a non-invasive technique, TEM thereby have potential to offer valuable predictive insights into the outcomes of invasive ART procedures. Although this finding requires further validation with direct ART outcome data, it indeed highlights the potential of TEM in personalized diagnostic and therapeutic planning, as well as its substantial value in clinical accessibility.

Nevertheless, our study has several limitations. Clinical reproductive outcomes (such as ART success rates), sperm functional parameters, and cost-effectiveness analyses were not included, thereby reducing the clinical/translational value of our findings. Additionally, the absence of longitudinal investigations (such as clinical reproductive outcomes) limits the ability to assess causal relationships, which is an inherent limitation of a cross-sectional design. Moreover, incomplete national coverage may have introduced selection bias. Future researches should therefore address these aspects to further supplement nationwide data, elucidate the association of ultrastructural defects with ART outcome, and subsequently establish diagnostic and therapeutic targets with more detailed standards.

## Supplementary Information

Below is the link to the electronic supplementary material.

Supplementary Material 1

## Data Availability

The datasets used and/or analyzed during the current study are available from the corresponding author on reasonable request.

## Abbreviations

ALH: Amplitude of lateral head displacement
ART: Assisted reproductive technique
BAAM: Both abnormality in axonemes and mitochondria
BCF: Beat cross frequency
BMI: Body mass index
CASA: Computer automated semen analysis system
ICSI: Intracytoplasmic sperm injection
IVF: In vitro fertilisation
LIN: Linearity
MAD: Mean angular displacement
NSM: Normal sperm morphology
NU: Normal ultrastructure
PSM: Progressive sperm motility
SAA: Simple abnormal axonemes
SAM: Simple abnormal mitochondria
SC: Sperm concentration
STR: Straightness
SV: Semen volume
TEM: Transmission electron microscopy
TSC: Total sperm count
TSM: Total sperm motility
VAP: Velocity average path
VCL: Velocity curvilinear
VSL: Velocity straight line
WOB: Wobble
