# Supplementary material for: Analysis of ultrastructural defects in sperm by transmission electron microscopy in asthenozoospermia patients: a study from multiple centers across China
Source: Biomark Res. 2025 Oct 8;13:122. doi: 10.1186/s40364-025-00841-8 (PMC12506306; doi:10.1186/s40364-025-00841-8)
Supplement: Supplementary file 1 — Supplementary Material 1 [file 40364_2025_841_MOESM1_ESM.docx]

**Supplementary materials**

**Table S1 Baseline Characteristics and Sperm Parameters of patients.**

| Variable | | | Fertile group  **(N=33)** | Asthenozoospermia group  **(N=106)** | ***P*** value |
| --- | --- | --- | --- | --- | --- |
| Baseline Characteristics | | | | | |
|  | Age (year, mean ± SD) | | 31.563 ± 4.826 | 33.210 ± 5.281 | 0.119 |
|  | BMI (mean ± SD) | | 24.678 ± 3.057 | 24.144 ± 2.911 | 0.455 |
|  | Ethnicity [N (percentage)] | | | | |
|  |  | Han | 33 (100%) | 100 (94.3%) | 0.162 |
|  |  | Non-Han | 0 (0%) | 6 (5.7%) |  |
|  | Education [N (percentage)] | | | | |
|  |  | Primary school | 2 (6.1%) | 17 (16.0%) | 0.275 |
|  |  | High school | 15 (45.5%) | 37 (34.9%) |  |
|  |  | College/University | 16 (48.4%) | 52 (49.1%) |  |
|  | Region [N (percentage)] | | | | |
|  |  | Northeastern | 11 (33.3%) | 31 (29.2%) | 0.506 |
|  |  | Southeastern | 6 (18.2%) | 31 (29.2%) |  |
|  |  | Southwestern | 3 (9.1%) | 13 (12.4%) |  |
|  |  | Northwestern | 13 (39.4%) | 31 (29.2%) |  |
|  | Poison exposure [N (percentage)] | | | | |
|  |  | Yes | 32 (97.0%) | 102 (96.2%) | 0.841 |
|  |  | No | 1 (3.0%) | 4 (3.8%) |  |
|  | Smoke [N (percentage)] | | | | |
|  |  | Yes | 28 (84.8%) | 86 (81.1%) | 0.627 |
|  |  | No | 5 (15.2%) | 20 (18.9%) |  |
|  | Alcohol [N (percentage)] | | | | |
|  |  | Yes | 30 (90.9%) | 95 (89.6%) | 0.830 |
|  |  | No | 3 (9.1%) | 11 (10.4%) |  |
|  | Drug abuse [N (percentage)] | | | | |
|  |  | Yes | 31 (93.9%) | 105 (99.1%) | 0.077 |
|  |  | No | 2 (6.1%) | 1 (0.9%) |  |
| **Conventional Sperm Parameters (mean ± SD)** | | | | | |
|  | SV (mL) | | 4.165 ± 1.839 | 3.746 ± 1.871 | 0.375 |
|  | SC (million/mL) | | 76.306 ± 44.223 | 33.066 ± 32.412 | <0.001* |
|  | TSC (million) | | 325.326 ± 276.080 | 152.140 ± 196.498 | 0.005* |
|  | TSM (%) | | 68.740 ± 9.905 | 26.315 ± 13.850 | <0.001* |
|  | PSM (%) | | 58.197 ± 9.702 | 16.135 ± 10.619 | <0.001* |
|  | NSM (%) | | 4.310 ± 2.121 | 3.372 ± 2.777 | 0.598 |
| Kinematic Sperm Parameters (mean ± SD) | | | | | |
|  | VCL (μm/s) | | 58.757 ± 11.701 | 34.469 ± 18.642 | <0.001* |
|  | VSL (μm/s) | | 39.220 ± 21.334 | 22.576 ± 16.815 | 0.001* |
|  | VAP (μm/s) | | 42.314 ± 8.689 | 24.250 ± 13.149 | <0.001* |
|  | ALH (μm) | | 4.188 ± 1.133 | 2.526 ± 1.860 | 0.001* |
|  | WOB (μm/s) | | 69.579 ± 8.881 | 57.716 ± 21.138 | 0.026* |
|  | BCF (μm/s) | | 15.343 ± 10.067 | 15.019 ± 11.072 | 0.913 |
|  | LIN (μm/s) | | 57.407 ± 11.195 | 46.353 ± 19.881 | 0.027* |
|  | MAD (°) | | 49.993 ± 4.559 | 64.582 ± 29.194 | 0.204 |
|  | STR (μm/s) | | 80.892 ± 7.608 | 69.831 ± 24.834 | 0.063 |

ALH, amplitude of lateral head displacement; BCF, beat cross frequency; LIN, linearity; BMI, body mass index; MAD, mean angular displacement; NSM, normal sperm morphology; PSM, progressive sperm motility; SC, sperm concentration; SD, standard deviation, STR, straightness; SV, semen volume; TSC, total sperm count; TSM, total sperm motility; VAP, velocity average path; VCL, velocity curvilinear; VSL, velocity straight line; WOB, wobble. *: *P*<0.05.

**Table S2 Ultrastructural performance of patients.**

| **Variable**  **[N (percentage)]** | | | | **Fertile group**  **(N=33)** | **Asthenozoospermia group**  **(N=106)** | ***P* value** |
| --- | --- | --- | --- | --- | --- | --- |
| **Head** | | | | | | |
|  | Shape | | | | | |
|  |  | Normal | | 18 (54.5%) | 55 (51.9%) | 0.789 |
|  |  | Abnormal **^#1^** | | 15 (45.5%) | 51 (48.1%) |  |
|  | Acrosomes | | | | | |
|  |  | Present | | 4 (12.1%) | 15 (14.2%) | 0.767 |
|  |  | Absent | | 29 (87.9%) | 91 (85.8%) |  |
|  | Chromatin | | | | | |
|  |  | Normal | | 21 (63.6%) | 37 (34.9%) | 0.003* |
|  |  | Abnormal **^#2^** | | 12 (36.4%) | 69 (65.1%) |  |
| **Tail** | | | | | | |
|  | Neck | | | | | |
|  |  | Cytoplasmic residues | | | | |
|  |  |  | Present | 24 (72.7%) | 93 (87.7%) | 0.039* |
|  |  |  | Absent | 9 (27.3%) | 13 (12.3%) |  |
|  |  | Centriole | | | | |
|  |  |  | Present | 19 (57.6%) | 32 (30.2%) | 0.004* |
|  |  |  | Absent | 14 (41.4%) | 74 (69.8%) |  |
|  | Midpiece | | | | | |
|  |  | Axonemes | | | | |
|  |  |  | Normal | 24 (72.7%) | 49 (46.2%) | 0.008* |
|  |  |  | Abnormal **^#3^** | 9 (27.3%) | 57 (53.8%) |  |
|  |  | Dense fibers | | | | |
|  |  |  | Normal | 24 (72.7%) | 46 (43.4%) | 0.003* |
|  |  |  | Abnormal **^#4^** | 9 (27.3%) | 60 (56.6%) |  |
|  |  | Mitochondria | | | | |
|  |  |  | Normal | 19 (57.6%) | 22 (20.8%) | <0.001* |
|  |  |  | Abnormal **^#5^** | 14 (41.4%) | 84 (79.2%) |  |
|  | Principal piece | | | | | |
|  |  | Axonemes | | | | |
|  |  |  | Normal | 20 (60.6%) | 27 (25.5%) | <0.001* |
|  |  |  | Abnormal **^#3^** | 13 (39.4%) | 79 (74.5%) |  |
|  |  | Dense fibers | | | | |
|  |  |  | Normal | 21 (63.6%) | 52 (49.1%) | 0.143 |
|  |  |  | Abnormal **^#4^** | 12 (36.4%) | 54 (50.1%) |  |
|  |  | Fibrous sheath | | | | |
|  |  |  | Present | 33 (100%) | 105 (98.1%) | 0.763 |
|  |  |  | Absent | 0 (0%) | 1 (1.9%) |  |
|  | Endpiece | | | | | |
|  |  | Axonemes | | | | |
|  |  |  | Normal | 12 (36.4%) | 10 (9.4%) | <0.001* |
|  |  |  | Abnormal **^#3^** | 21 (63.6%) | 96 (90.6%) |  |

#1: abnormal shape of head contained multi-nucleated head and altered shape of head showed in Fig. 1B.

#2: abnormal chromatin contained severe vacuolar defect of the chromatin and granular chromatin showed in Fig. 1B.

#3: abnormal axonemes contained abnormal quantity of axonemes as well as translocation or misassembled of axonemes showed in Fig. 1C.

#4: abnormal dense fibers contained abnormal quantity of axonemes as well as disordered arrangement or translocation of dense fibers showed in Fig. 1C.

#5: abnormal mitochondria contained abnormal quantity and abnormal morphology of mitochondria showed in Fig. 1C.

*: *P*<0.05

**Table S3 The specific grouping criteria for subgroups.**

|  | | | **Normal ultrastructure (NU)** | **Simple abnormal axonemes (SAA)** | **Simple abnormal mitochondria (SAM)** | **Both abnormality in axonemes and mitochondria (BAAM)** |
| --- | --- | --- | --- | --- | --- | --- |
| **Head** | | | | | | |
|  | Shape | | Not application | | | |
|  | Acrosomes | | Not application | | | |
|  | Chromatin | | Not application | | | |
| **Tail** | | | | | | |
|  | Neck | | | | | |
|  |  | Cytoplasmic residues | Not application | | | |
|  |  | Centriole | Not application | | | |
|  | **Midpiece** | | | | | |
|  |  | **Axonemes & Dense fibers** | **Normal** | **Abnormal** | **Normal** | **Abnormal** |
|  |  | **Mitochondria** | **Normal** | **Normal** | **Abnormal** | **Abnormal** |
|  | Principal piece^#^ | | | | | |
|  |  | Axonemes & Dense fibers | Not application | | | |
|  |  | Fibrous sheath | Not application | | | |
|  | Endpiece^#^ | | | | | |
|  |  | Axonemes | Not application | | | |

#: The performance of Principal piece and Endpiece were almost exactly the same as the performance of Midpiece, so only the performance of Midpiece was observed in the present study.

**Table S4 Detailed summary of the relevant previous researches on the associations between the various ultrastructural defects of sperm and the outcome of ICSI.**

| **Number** | **Author (Year)** | **Research object** | **Sample size** | **Country** | **Ultrastructural defects in sperm** | | | | **Impact of ultrastructural defects on ICSI ^#1^** | |
| --- | --- | --- | --- | --- | --- | --- | --- | --- | --- | --- |
|  |  |  |  |  | **Flagella** | | **Mitochondria** | |  |  |
| **Definite impact of ultrastructural defects on ICSI** | | | | | | | | | | |
| 1 | Mingrong Lv (2022) (1) | Potassium channel subfamily U1 (*KCNU1*, also known as *SLO3*) variants | 1 patient with controls | China | **×** | No obvious defects in the ultrastructure of the flagella. | **√** | A seriously disorganized or short MS. | **√** | An impact on the clinical pregnancy rate. |
| 2 | Lucile Ferreux (2021) (2) | Sperm ultrastructural defects observation | 25 | France | **√** | Absence of axonemes (CP). | **×** | -^#2^ | **×** | No impact on the live birth rate. |
| 3 | Angèle Boursier (2023) (3) | Sperm flagellum ultrastructural defects observation | 177 | France | **√** | Thick or disorganized fibrous sheath, a lack of inner/outer dynein arm, hemi-axonemes, lack of microtubular pair and microtubular translocations. | **×** | -^#2^ | **×** | No impact on the fertilization rate, the occurrence of a cumulative pregnancy, the clinical pregnancy rate, and the live birth rate. |
| **The outcome of ICSI is reported, however, no comparison of the the outcome of ICSI is performed between case and control groups** | | | | | | | | | | |
| 4 | Chen Tan (2021) (4) | Dynein heavy chain domain 1 *(DNHD1)* variants | 8 patients with controls | China | Absence of CP and disorganization of axonemes. | | Poorly assembled MS. | | 4/7 successful pregnancy.^#3^ | |
| 5 | Yang Gao (2021) (5) | Dynein axonemal heavy chain 2 (*DNAH2)* variants | 3 patients with controls | China | Defects in axonemes (CP) | | -^#2^ | | 2/2 successful clinical pregnancy.^#3^ | |
| 6 | Fei Huang (2023) (6) | Dynein axonemal heavy chain 6 (*DNAH6)* gene | 1 patient with controls | China | Serious distortions in axonemes. | | -^#2^ | | 1/1 successfully fertilized.^#3^ | |
| 7 | Zhong-Mei Shao (2023) (7) |  | 3 patients with controls | China | Absence of axonemes (CP), defects in radial spokes. | | A proportion of the disordered MS. | | 1/1 give birth to a healthy child.^#3^ | |
| 8 | Yanwei Sha (2023) (8) | Adenylate kinase 9 (*AK9*) variants | 5 patients with controls | China | No obvious defects in the ultrastructure of the flagella. | | -^#2^ | | 3/3 give birth to a healthy child.^#3^ | |
| 9 | Wensheng Liu (2023) (9) | Armadillo repeat (ARM-repeat) family - *ARMC12* variants | 3 patients with controls | China | Scattered or forked axonemes. | | Loss of mitochondria. | | 2/2 give birth to a healthy child.^#3^ | |
| 10 | Célia Tebbakh (2025) (10) | nexin-dynein regulatory complex (N-DRC) family - *DRC1* variants | 4 patients with controls | France | An incomplete axonemes with the dissociation of the peripheral doublets. | | Rare hypertrophic mitochondria. | | 3/4 give birth to a healthy child.^#3^ | |
| 11 | Zhihua Zhang (2023) (11) | IQ motif and ubiquitin-like domain-containing (*IQUB*) variants | 1 patient with controls | China | Defects in radial spoke. | | -^#2^ | | 1/1 successfully fertilized.^#3^ | |
| 12 | Shengjia Shi (2024) (12)  &  Yaqian Li (2021) (13) | Leucine-rich repeat containing 6 (*LRRC6*) variants | 1 patient with controls  &  1 patient with controls | China | A lack of outer/inner dynein arms. | | An incomplete MS surrounding the axonemes. | | 1/1 give birth to a healthy child.^#3^ | |
| 13 | Song-Xi Tang (2024) (14) | Leucine-rich repeat containing 23 (*LRRC23*) variants | 1 patient with controls | China | Radial spoke defects. | | -^#2^ | | 1/1 give birth to a healthy child.^#3^ | |
| 14 | Feng Wan (2023) (15) | Dynein axonemal assembly factor 3 (*DNAAF3*) variants | 1 patient with controls | China | A lack of outer/inner dynein arms. | | -^#2^ | | 1/1 give birth to a healthy child.^#3^ | |
| 15 | Weili Wang (2023) (16) | Potassium Channel Tetramerization Domain Containing 19 (*KCTD19*) variants | 3 patients with controls | China | Disorganized flagella components. | | -^#2^ | | 1/3 give birth to a healthy child.^#3^  Compared with other studies, the lower live birth rate in this study may result from significant abnormalities in the sperm heads. | |
| 16 | Jiangshan Cong (2022) (17) | *CCDC34* variants | 1 patient with controls | China | Disordered dense fibres, and abnormal axonemes. | | -^#2^ | | 72.7% fertilization for this patient.^#3^ | |
| 17 | Chuan Xu (2022) (18) | sperm-associated antigen 6 (*SPAG6)* variants | 2 patients with controls | China | Defects in flagella (mainly lack of CP). | | -^#2^ | | 1/1 successfully conceived.^#3^ | |
| 18 | Gan Shen (2025) (19) | cilia and flagella associated protein (CFAP) family -*CFAP221* variants | 2 patients with controls | China | Defects in axonemes. | | -^#2^ | | 2/2 successful clinical pregnancy.^#3^ | |
| **ICSI is not mentioned** | | | | | | | | | | |
| 19 | Guillaume Martinez (2023) (20) | Dynein heavy chain domain 1 *(DNHD1)* variants | 3 | Iran and Europe | An absence of CP, an abnormal number of dense fibers, and completely disorganized of flagella. | | Poorly assembled and misshapen MS. | | **-**^#2^ | |
| 20 | Jae Yeon Hwang (2021) (21) | Dynein axonemal heavy chain 2 (*DNAH2)* variants | 1 patient with controls | Pakistan | Misaligned dense fiber, missing fibrous sheath, and incomplete axonemes. | | Abnormally arranged mitochondria. | | -^#2^ | |
| 21 | Sobia Dil (2023) (22) | Dynein axonemal heavy chain 8 (*DNAH8)* variants | 2 patients with controls | Pakistan | Disorganized or missing peripheral microtubule doublets and dense fibers and absent CP. | | -^#2^ | | -^#2^ | |
| 22 | Fei Yan (2024) (23) | Dynein axonemal heavy chain 9 (*DNAH9)* variants | 2 patients with controls | China | Lack of outer dynein arms. | | -^#2^ | | -^#2^ | |
| 23 | Beibei Zhang (2021) (24) | Dynein axonemal heavy chain 17 (*DNAH17)* variants | 3+1 | Pakistani + China | Defects in axonemes. | | -^#2^ | | -^#2^ | |

CP: central pair of microtubules, ICSI: intracytoplasmic sperm injection, MS, mitochondrial sheath.

#1: Not all the participants undergo ICSI.

#2: Not mentioned.

#3: The outcome of ICSI is reported, however, no comparison of the the outcome of ICSI is performed between case and control groups.

**Figure S1. Map of China showing study locations.**


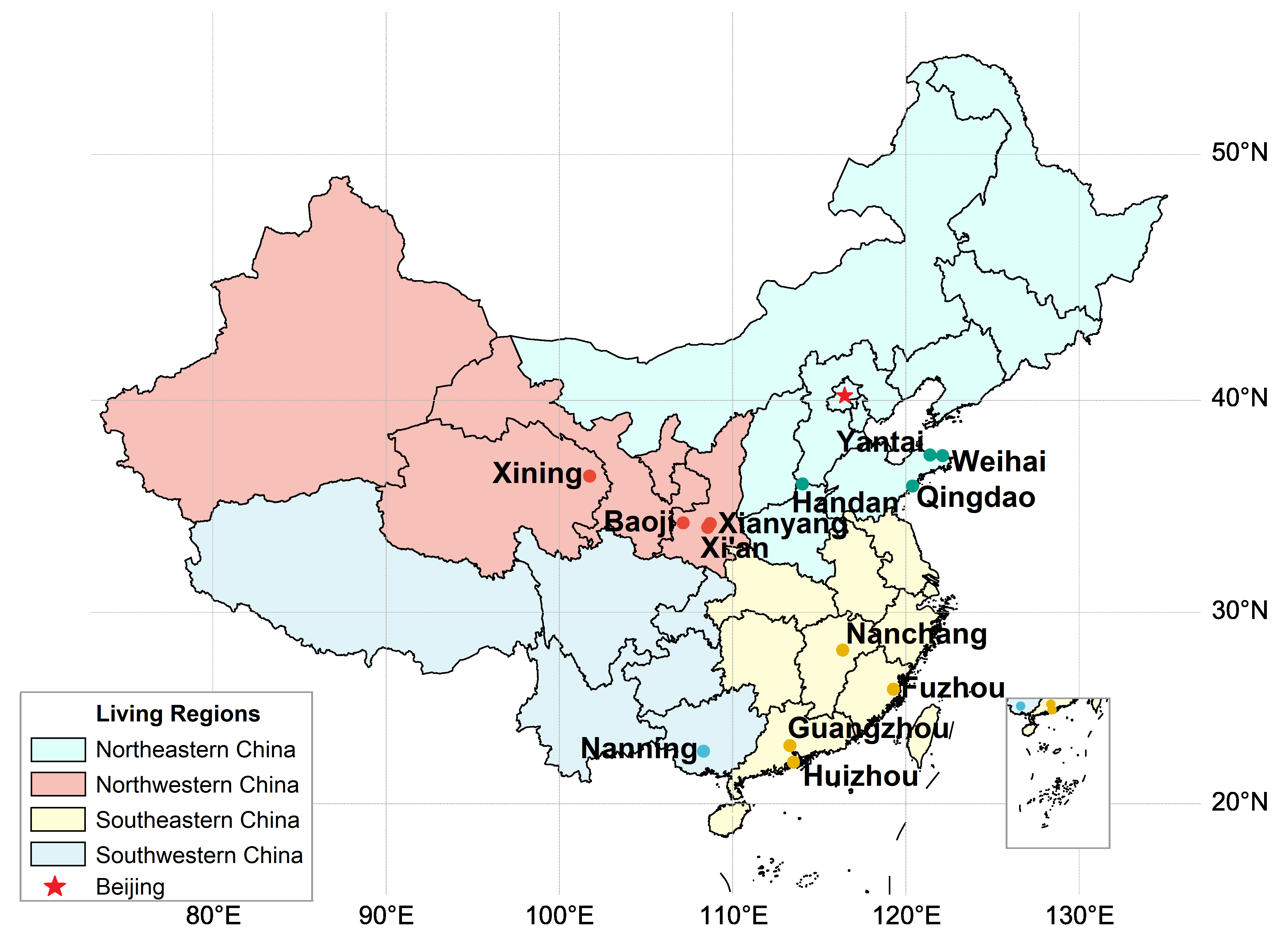


**Figure S2. Correlations of ultrastructural performances^#^ with baseline characteristic**s **and sperm parameters.**


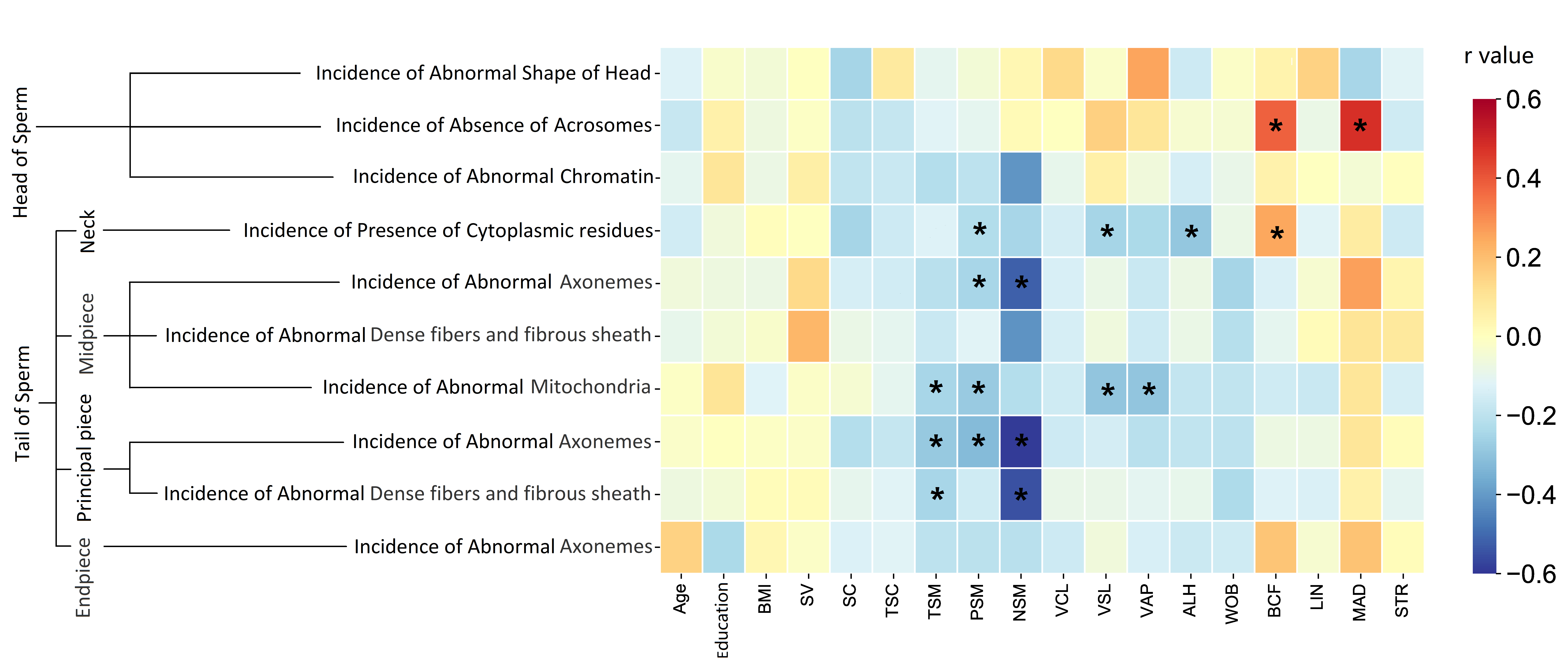


#: Incidence of absence of fibrous sheath was not included in the correlation analysis due to the insufficient data (only one sample exhibited the absence of fibrous sheath).

ALH, amplitude of lateral head displacement; BCF, beat cross frequency; BMI, body mass index; LIN, linearity; MAD, mean angular displacement; NSM, normal sperm morphology; PSM, progressive sperm motility; SC, sperm concentration; STR, straightness; SV, semen volume; TSC, total sperm count; TSM, total sperm motility; VAP, velocity average path; VCL, velocity curvilinear; VSL, velocity straight line; WOB, wobble. *: *P*<0.05.

**Supplementary methods**

**Study Population**

Asthenozoospermia patients were randomly recruited to the study from February 2022 to December 2022. The diagnosis of asthenozoospermia was determined according to the criteria of the 6^th^ World Health Organization (WHO) guidelines (25), which was total sperm motility (TSM) <42% or progressive sperm motility (PSM) <30%. Results should be confirmed by two or three semen analyses. Normal fertile donors who had fathered no less than one child in the past 12 months and had normal sperm parameter values were recruited as the controls. Participants with history of karyotype abnormalities, chronic diseases, pelvic/spinal injuries, and microdeletions of azoospermia factor (AZF) region on Y chromosome were also excluded. In addition, during period of data collection, Chinese hospitals have not yet achieved full coverage of the new 6^th^ WHO guidelines, and some hospitals still follow the old 5^th^ WHO guidelines (26). Hence, data from centers using the 5^th^ and 6^th^ WHO guidelines were included, on the contrary, data from centers using more earlier version of WHO guidelines or not mentioned to following WHO guidelines were excluded. Moreover, since variation of the standard reference values of the 5^th^ and 6^th^ guidelines are slightly different, in order to achieve standardization across all centers, all participants undergo diagnosis based on 6^th^ WHO guidelines, no matter his original center used the 5^th^ or 6^th^ WHO guidelines.

G-Power was applied for sample size calculation. In the Chi-square test, the effect size was set to 0.3, following Cohen's guidelines with α = 0.05 and power = 0.9 (27). The degree of freedom (Df) was calculated using the formula: Df = (R - 1) x (C - 1), where R represented the number of rows (2, Fertile group or Asthenozoospermia group) and C represented the number of columns (2, Normal/Present or Abnormal/Absent). Substituted these values, Df was calculated as 1. Based on these parameters, G-power indicated a minimum sample size of 117. To account for potential sampling errors, a 10% buffer was applied, resulting in a final sample size of 130 participants.

This study was approved by Ethics Committee of Guangzhou Huayin Medical Testing Center (No. LLPJ2022002). The purpose of this study was explained to each patient, and written informed consent was obtained from all subjects prior to enrolment.

**Questionnaire and Physical Examination**

Questionnaires collected the baseline characteristics, containing age, ethnicity, education level, living region, poison exposure, smoking status, alcohol consumption, and drug abuse, and were completed by each participant. Additionally, physical examinations were also conducted including measurement of body mass index (BMI) and genital examination.

**Sperm assay**

The sperm assay was performed as previously described (28). Specifically, the semen samples were obtained after 3-7 days of abstinence, and then was assessed according to the 6^th^ WHO guidelines (25). Five conventional sperm parameters, including semen volume (SV), sperm concentration (SC), total sperm count (TSC), TSM and PSM, and nine kinematic sperm parameters, including velocity curvilinear (VCL), velocity straight line (VSL), velocity average path (VAP), amplitude of lateral head displacement (ALH), wobble (WOB), beat cross frequency (BCF), linearity (LIN), mean angular displacement (MAD), and straightness (STR), were assessed by a well-trained technician using the computer automated semen analysis system (CASA, WLJY-9000, China). In addition, normal sperm morphology (NSM) was determined using smears after staining with the Baso Papanicolaou staining method.

**Transmission Electron Microscopy**

Semen samples were selected and transferred into the collecting tube and then combined with 2.5% gluteraldehyde in 0.1 mol/L sodium cacodylate/HCl buffer, pH 7.2-7.4, for 4-6 h at room temperature or overnight at 4°C. Thereafter, the specimen was centrifuged at 500 g for 5 min and the sperm pellet was postfixed in 1% osmium tetroxide for 2 h, dehydrated in ascending graded alcohol and embedded in epoxy resin. Blocks containing abundant sperm were identified via visual examination, and ultra-thin sections (60-80 nm thick) were cut. Sections were double-stained with uranyl acetate and lead citrate. Ultrathin sections were examined using an H7500 transmission electron microscope (Hitachi, Tokyo, Japan) at 2,500× - 50,000× magnification. Ultrastructure evaluated included head shape (normal or abnormal), acrosomes (present or absent), chromatin (normal or abnormal), neck cytoplasmic residues (present or absent), centriole (present or absent), axonemes (normal or abnormal), dense fibers (normal or abnormal), mitochondria (normal or abnormal), and fibrous sheath (present or absent). Single ultrastructural alterations were evaluated and counted with a minimum of 200 observations for each sample. All the counts were conducted by two independent operators, and the final value of each ultrastructure was the mean value of the two researchers. Additionally, according to the percentage of the abnormal ultrastructure, the abnormal groups were further divided into tripartite-stratified levels: mild (≤ 33%); moderate (34% - 66%); severe (≥ 66%).

**Statistical analysis**

Continuous data were expressed as mean ± standard deviation (SD). The non-normally distributed data were log-transformed before statistical analysis. The difference between two groups was evaluated by independent-samples t-test. Categorical data were expressed as frequencies of the total, and differences of distribution from different groups was evaluated by Chi-square test. The potential associations of ultrastructural performance with baseline characteristics and sperm parameters were determined by Spearman correlation test.

We used SPSS (V.18.0, SPSS Inc, IL, USA) for all analyses. All *P*-values were two-sided and *P*<0.05 was considered to be statistically significant.

**References:**

1. Lv M, Liu C, Ma C, Yu H, Shao Z, Gao Y, et al. Homozygous mutation in SLO3 leads to severe asthenoteratozoospermia due to acrosome hypoplasia and mitochondrial sheath malformations. Reproductive biology and endocrinology : RB&E 2022; 20: 5.

2. Ferreux L, Bourdon M, Chargui A, Schmitt A, Stouvenel L, Lorès P, et al. Genetic diagnosis, sperm phenotype and ICSI outcome in case of severe asthenozoospermia with multiple morphological abnormalities of the flagellum. Human reproduction (Oxford, England) 2021; 36: 2848-60.

3. Boursier A, Boudry A, Mitchell V, Loyens A, Rives N, Moerman A, et al. Results and perinatal outcomes from 189 ICSI cycles of couples with asthenozoospermic men and flagellar defects assessed by transmission electron microscopy. Reprod Biomed Online 2023; 47: 103328.

4. Tan C, Meng L, Lv M, He X, Sha Y, Tang D, et al. Bi-allelic variants in DNHD1 cause flagellar axoneme defects and asthenoteratozoospermia in humans and mice. Am J Hum Genet 2022; 109: 157-71.

5. Gao Y, Tian S, Sha Y, Zha X, Cheng H, Wang A, et al. Novel bi-allelic variants in DNAH2 cause severe asthenoteratozoospermia with multiple morphological abnormalities of the flagella. Reprod Biomed Online 2021; 42: 963-72.

6. Huang F, Zeng J, Liu D, Zhang J, Liang B, Gao J, et al. A novel frameshift mutation in DNAH6 associated with male infertility and asthenoteratozoospermia. Front Endocrinol (Lausanne) 2023; 14: 1122004.

7. Shao ZM, Zhu YT, Gu M, Guo SC, Yu H, Li KK, et al. Novel variants in DNAH6 cause male infertility associated with multiple morphological abnormalities of the sperm flagella (MMAF) and ICSI outcomes. Asian journal of andrology 2024; 26: 91-8.

8. Sha Y, Liu W, Li S, Osadchuk LV, Chen Y, Nie H, et al. Deficiency in AK9 causes asthenozoospermia and male infertility by destabilising sperm nucleotide homeostasis. EBioMedicine 2023; 96: 104798.

9. Liu W, Wei X, Liu X, Chen G, Zhang X, Liang X, et al. Biallelic mutations in ARMC12 cause asthenozoospermia and multiple midpiece defects in humans and mice. J Med Genet 2023; 60: 154-62.

10. Tebbakh C, Barbotin AL, Martinez G, Boursier A, Wehbe Z, Hammouda A, et al. A recurrent loss-of-function variant in DRC1 causes non-syndromic severe asthenozoospermia with favorable intracytoplasmic sperm injection and pregnancy outcomes. Andrology 2025.

11. Zhang Z, Zhou H, Deng X, Zhang R, Qu R, Mu J, et al. IQUB deficiency causes male infertility by affecting the activity of p-ERK1/2/RSPH3. Human reproduction (Oxford, England) 2023; 38: 168-79.

12. Shi S, Tang X, Long S, Yang J, Wang T, Wang H, et al. A novel homozygous LRRC6 mutation causes male infertility with asthenozoospermia and primary ciliary dyskinesia in humans. Andrology 2025; 13: 459-72.

13. Li Y, Jiang C, Zhang X, Liu M, Sun Y, Yang Y, et al. The effect of a novel LRRC6 mutation on the flagellar ultrastructure in a primary ciliary dyskinesia patient. Journal of assisted reproduction and genetics 2021; 38: 689-96.

14. Tang SX, Liu SY, Xiao H, Zhang X, Xiao Z, Zhou S, et al. Novel mutations in LRRC23 cause asthenozoospermia in a nonconsanguineous family. Asian journal of andrology 2024; 26: 484-9.

15. Wan F, Yu L, Qu X, Xia Y, Feng K, Zhang L, et al. A novel mutation in PCD-associated gene DNAAF3 causes male infertility due to asthenozoospermia. J Cell Mol Med 2023; 27: 3107-16.

16. Wang W, Su L, Meng L, He J, Tan C, Yi D, et al. Biallelic variants in KCTD19 associated with male factor infertility and oligoasthenoteratozoospermia. Human reproduction (Oxford, England) 2023; 38: 1399-411.

17. Cong J, Wang X, Amiri-Yekta A, Wang L, Kherraf ZE, Liu C, et al. Homozygous mutations in CCDC34 cause male infertility with oligoasthenoteratozoospermia in humans and mice. J Med Genet 2022; 59: 710-8.

18. Xu C, Tang D, Shao Z, Geng H, Gao Y, Li K, et al. Homozygous SPAG6 variants can induce nonsyndromic asthenoteratozoospermia with severe MMAF. Reproductive biology and endocrinology : RB&E 2022; 20: 41.

19. Shen G, Tian E, Jiang C, Tian Y, Zhang Y, Wang X, et al. Establishing the causative link between CFAP221 variants and asthenoteratozoospermia in humans. Journal of assisted reproduction and genetics 2025; 42: 1975-87.

20. Martinez G, Barbotin AL, Cazin C, Wehbe Z, Boursier A, Amiri-Yekta A, et al. New Mutations in DNHD1 Cause Multiple Morphological Abnormalities of the Sperm Flagella. Int J Mol Sci 2023; 24.

21. Hwang JY, Nawaz S, Choi J, Wang H, Hussain S, Nawaz M, et al. Genetic Defects in DNAH2 Underlie Male Infertility With Multiple Morphological Abnormalities of the Sperm Flagella in Humans and Mice. Front Cell Dev Biol 2021; 9: 662903.

22. Dil S, Khan A, Unar A, Yang ML, Ali I, Zeb A, et al. A novel homozygous frameshift variant in DNAH8 causes multiple morphological abnormalities of the sperm flagella in a consanguineous Pakistani family. Asian journal of andrology 2023; 25: 350-5.

23. Yan F, Zhi W, Wei Y, Dai L, Xu W, Zheng R. Novel variants in DNAH9 are present in two infertile patients with severe asthenospermia. J Hum Genet 2025; 70: 105-11.

24. Zhang B, Khan I, Liu C, Ma A, Khan A, Zhang Y, et al. Novel loss-of-function variants in DNAH17 cause multiple morphological abnormalities of the sperm flagella in humans and mice. Clin Genet 2021; 99: 176-86.

25. Organization WH. WHO laboratory manual for the examination and processing of human semen (6th edition). WHO Press 2021.

26. Organization WH. WHO laboratory manual for the examination and processing of human semen (5th edition). WHO Press 2010.

27. Cohen J. A power primer. Psychological Bulletin 1992; 112: 155-9.

28. Lv MQ, Wang HX, Yang YQ, Sun RF, Ge P, Zhang J, et al. Semen Quality Following Long-term Occupational Exposure to Formaldehyde in China. JAMA network open 2022; 5: e2230359.
